# Blood Group Antigens C, Lub and P1 May Have a Role in HIV Infection in Africans

**DOI:** 10.1371/journal.pone.0149883

**Published:** 2016-02-22

**Authors:** Modisa Sekhamo Motswaledi, Ishmael Kasvosve, Oluwafemi Omoniyi Oguntibeju

**Affiliations:** 1 Department of Medical Laboratory Sciences, Faculty of Health Sciences, University of Botswana, Gaborone, Botswana; 2 Nutrition & Chronic Diseases Research Group, Oxidative Stress Research Centre, Department of Biomedical Sciences, Faculty of Health & Wellness Sciences, Cape Peninsula University of Technology, Cape Town, South Africa; University of Cincinnati College of Medicine, UNITED STATES

## Abstract

**Background:**

Botswana is among the world’s countries with the highest rates of HIV infection. It is not known whether or not this susceptibility to infection is due to genetic factors in the population. Accumulating evidence, however, points to the role of erythrocytes as potential mediators of infection. We therefore sought to establish the role, if any, of some erythrocyte antigens in HIV infection in a cross-section of the population.

**Methods:**

348 (346 HIV-negative and 2 HIV-positive) samples were obtained from the National Blood Transfusion Service as residual samples, while 194 HIV-positive samples were obtained from the Botswana-Harvard HIV Reference Laboratory. Samples were grouped for twenty three antigens. Chi-square or Fischer Exact analyses were used to compare the frequencies of the antigens in the two groups. A stepwise, binary logistic regression was used to study the interaction of the various antigens in the light of HIV-status.

**Results:**

The Rh antigens C and E were associated with HIV-negative status, while blood group Jk^a^, P_1_ and Lu^b^ were associated with HIV-positive status. A stepwise binary logistic regression analysis yielded group C as the most significant protective blood group while Lu^b^ and P_1_ were associated with significantly higher odds ratio in favor of HIV-infection. The lower-risk-associated group C was significantly lower in Africans compared to published data for Caucasians and might partially explain the difference in susceptibility to HIV-1.

**Conclusion:**

The most influential antigen C, which also appears to be protective, is significantly lower in Africans than published data for Caucasians or Asians. On the other hand, there appear to be multiple antigens associated with increased risk that may override the protective role of C. A study of the distribution of these antigens in other populations may shed light on their roles in the HIV pandemic.

## Introduction

A number of investigators have alluded to the role of blood groups in HIV epidemiology. For example, Arendrup and colleagues reported that HIV from lymphocytes of blood group A individuals was neutralized by anti-A, implying that this mechanism could potentially reduce the likelihood of infection in ABO discordant couples [1]. These findings were later corroborated by Neil and colleagues [2]. Furthermore, other investigators have reported higher prevalence of HIV-2 in blood group such as AB [3], O [4] as well as a protective function associated with blood group P^k^ [5, 6]. However, all these studies focused on a small number of erythrocyte antigens.

Genetic factors other than blood groups have been shown to influence susceptibility to HIV infection and disease progression. Individuals with a double deletion of 32 base pairs in the CCR5 (CCR5∆32/∆32) molecule and other mutants of the same gene are known to be resistant to HIV infection with R5 viruses [7, 8]. A virtual cure for HIV has thus been attained in one patient following bone marrow replacement with this cell line[9]. Furthermore, genetic composition of major histocompatibility complex class I (MHC-I) have been documented to influence HIV susceptibility[10]. In this regard, being heterozygous for some MHC-I alleles is thought to provide greater variety for antigen presentation to cytotoxic T cells and therefore more efficacy at clearing virally-infected CD4^+^ cells[11]. Moreover, some mutations in MHC-II molecules have been shown to promote humoral[12] response or enhance the effect of Natural Killer cells[13], giving an overall effect of enhanced resistance to HIV infection and disease progression. While this list is not exhaustive, it suffices to indicate that additional molecules could exist that could potentially influence susceptibility to HIV infection and disease progression. This study was motivated by increasing reports of HIV interaction with erythrocytes and the potential for their surface molecules in mediating the infection of CD4^+^ cells. Many studies have demonstrated the promiscuity of red blood cell antigens in binding to bacterial toxins, viruses and other molecules [14–17]. The Duffy antigen receptor for chemokines (DARC) is the receptor for *Plasmodium vivax [18, 19]* and has stimulated debate on its role in HIV-infection as a candidate for HIV-binding and infecting susceptible cells [17, 20–23]. Other reports have suggested that certain erythrocyte lipids promote viral membrane fusion with CD4^+^ cells and thus facilitate infection [24–26]. The predilection of HIV for any particular blood group could therefore explain the epidemiology among populations expressing that antigen to varying degrees.

Botswana is one of the countries in the world most hard-hit by the HIV pandemic with estimated national prevalence of 17.6%[27] and as high as 33.7% among pregnant women aged 15–49 years[28]. While risky behaviors have been documented in this population[29], it is not known whether the susceptibility of this population was purely attributable to its social habits or whether there exists a genetic predisposition to HIV infection that exacerbated its vulnerability. If indeed erythrocyte surface molecules bind and increase the transfection of CD4^+^ cells, we hypothesize that this should reflect in the prevalence of HIV associated with those erythrocyte surface molecules. Blood groups are probably the most abundant molecules on the red blood cell surface and are therefore logical candidates for this investigation. We thus sought to find out if the presence of any particular blood group antigen could be linked to increased HIV infection risk in Botswana. Our results suggest Rh blood group C to be protective and the other groups Lu^b^ and P_1_ to increase the odds ratio in favor of HIV infection.

## Materials and Methods

### Study Population

Three hundred and forty six (346) HIV-negative blood samples and two (2) HIV-positive samples were obtained from the National Blood Transfusion Service (NBTS) as residual samples. The National Blood Transfusion Service is the main center for donor recruitment as well as screening for suitability of blood donors and for ensuring the safety of donated blood and its components. One hundred and ninety four (194) HIV-positive samples were obtained from the Botswana-Harvard HIV Reference Laboratory (BHHRL). This laboratory is a referral laboratory in the management of HIV/AIDS patients and functions mainly to support the anti-retroviral treatment program by providing CD4 counts and viral load testing services for prospective and ARV-enrolled patients. All samples from the ARV-enrolled patients were obtained as unlabeled aliquots and assigned new study identification numbers. HIV-positive samples included both new cases and those from patients already on anti-retroviral therapy. For NBTS samples, donor identification numbers, not names, were retained only until HIV results were entered, at which point donor numbers were removed and replaced with new study numbers. The total number of samples tested for each blood group was determined by the availability of the specific antiserum taking into consideration the sample size of similar studies in published works.

Ethical approval was obtained prior to commencement of the study from the Ethics Committee of the University of Botswana under the Office of Research and Development, Princess Marina Hospital Research Committee, and from the Research Ethics Committee of the Faculty of Health and Wellness Sciences at Cape Peninsula University of Technology. Informed consent was not necessary since the research utilized residual samples that were rendered anonymous in accordance with the requirements of ISO 15189 [30].

### Determination of Blood Groups

Patient and donor red cells were phenotyped using specific antisera and following routine tube-grouping technique as outlined in the manufacturer’s instructions for agglutinating and non-agglutinating antibodies (Fortress Diagnostics, Antrim, United Kingdom). The strength of the reactions was graded on a scale of 0–4, where 0 represented no agglutination, 1+ represented small agglutinates in a suspension of free cells, 2+ being small agglutinates with a clear background, 3+ being several large agglutinates with a clear background and 4+ representing a single large agglutinate with a clear background. The manufacturer’s instructions were varied for Kidd antibodies (anti-Jk^a^ and anti-Jk^b^) since they only passed quality control when subjected to the manufacturer’s procedure for non-agglutinating antibodies. It was reasonable to subject them to this procedure since they are known to be non-agglutinating. The anti-human globulin (AHG) procedure was used to determine the reactivity of the following antibodies: anti-Fy^a^, anti-Fy^b^ anti-Jk^a^, anti-Jk^b^, anti-Kp^a^, anti-Kp^b^, anti-Lu^a^, anti-Lu^b^, Anti-S and anti-s. The AHG was also used to confirm negative results for anti-D. The specificity and reactivity of the antisera was confirmed by use of an antibody identification panel, DiaPanel^®^, Lot 16211.66.x-16311.66.x (BioRad^®^, Japan). The blood antigens tested for in this study included: A, B, “O”, D, C, c, E, e, Fy^a^, Fy^b^, Jk^a^, Jk^b^, Kp^a^, Kp^b^, Le^a^, Le^b^, Lu^a^, Lu^b^, M, N, P_1_, S, and s.

### Statistical Analysis

Data analysis was conducted using IBM SPSS version 23 software. Comparison of proportions of blood group antigens between HIV-infected and non-infected individuals were conducted using Pearson’s chi-square test (χ2) or Fisher’s exact test where appropriate. We explored the combined effect of various blood groups on HIV prevalence using a stepwise binary logistic regression analysis. Only blood group antigens with statistically significant association with HIV status in bivariate analyses were included in the model and the results were reported as odds ratio with 95% confidence interval. Results were considered significant at p < 0.05.

## Results

In total, 348 HIV-negative blood samples and 196 blood samples from HIV-infected patients were studied. The results comparing the prevalence of each antigen in the HIV group versus uninfected controls are summarized in **Table 1**. The frequency of C Rh antigen was lower in HIV-infected patients than in HIV-negative subjects, 17.1% (95% CI 10.7–23.5) versus 29.7% (95% CI 24.1–35.1), respectively p = 0.006. Similarly, the E Rh antigen was lower in HIV-infected patients than in HIV-negative subjects, 12.1% (95% CI 6.6–17.6) versus 21.2% (95% CI 16.2–26.2), respectively p = 0.024. Only these Rh-antigens raised the prospects of a protective function against HIV-1 infection.

**Table 1:** Comparison of prevalence of blood group antigen in HIV-positive patients and HIV-negative subjects.

Antigen	HIV-positive Subjects		HIV-negative Subjects		P-value
	N	Antigen Frequency % (95% CI)	N	Antigen Frequency % (95% CI)	
A	155	34.1 (26.5–41.7)	239	42.3 (35.9–48.7)	0.109
B	155	32.3 (24.8–39.8)	239	25.9 (20.2–31.6)	0.174
O	239	41.3 (47.3–65.9)	336	41.0 (35.6–46.4)	1
D	155	95.5 (92.2–98.8)	239	97.1 (94.9–99.3)	0.406
C	140	17.1 (10.7–23.5)	269	29.7 (24.1–35.3)	0.006
c	140	99.3 (97.9–100.7)	269	97.4 (95.5–99.3)	0.273
E	140	12.1 (6.6–17.6)	269	21.2 (16.2–26.2)	0.024
e	140	99.3 (97.9–100.7)	269	98.5 (97.0–100)	0.665
Fy^a^	146	10.3 (5.3–15.3)	251	9.6 (5.9–13.3)	0.818
Fy^b^	146	12.3 (6.9–17.7)	254	9.8 (6.1–13.5)	0.44
Jk^a^	116	85.3 (78.7–91.9)	109	51.4 (41.8–61.0)	<0.0001
Jk^b^	117	25.6 (17.5–33.7)	109	31.2 (22.3–40.1)	0.355
Kp^a^	123	2.5 (-0.3–5.3)	264	1.1 (-0.2–2.4)	0.387
Kp^b^	97	45.4 (35.3–55.5)	266	36.5 (30.6–42.4)	0.124
Le^a^	82	30.5 (20.3–40.7)	257	25.3 (19.9–30.7)	0.354
Le^b^	66	50.0 (37.7–62.3)	257	42.0 (35.8–48.2)	0.244
Lu^a^	118	1.7 (-0.7–4.1)	266	3.8 (1.5–6.1)	0.358
Lub	113	86.7 (80.3–93.1)	240	67.8 (61.8–73.8)	0.0001
M	92	77.2 (68.5–85.9)	236	80.9 (75.8–86.0)	0.456
N	93	71.0 (61.6–80.4)	269	66.9 (61.2–72.6)	0.47
P_1_	105	89.2 (83.1–95.3)	248	74.6 (69.1–80.1)	0.003
S	125	33.6 (25.2–42.0)	225	31.6 (25.4–37.8)	0.695
s	111	95.6 (91.7–99.5)	226	89.4 (85.3–93.5)	0.06

In contrast, the HIV-infected group exhibited a significantly higher prevalence of blood groups Jk^a^, Lu^b^ and P_1_ compared to HIV-negative individuals, suggesting a potential infection risk associated with carriage of these antigens. Among the HIV-infected patients the prevalence of Jk^a^ was 85.3% ((95% CI 78.7–91.9) compared to 51.4% (95% CI 41.8–61.0) in non-infected individuals, p = 0.024. The prevalence of Lu^b^ was 86.7% (95% CI 80.3–93.1) among HIV patients versus 67.8% (95% CI 61.8–73.8) in non-HIV infected subjects, p = 0.0001. Among the HIV-infected patients, P_1_ prevalence was 89.2% (95% CI 83.1–95.3) compared to 74.6% (95% CI 69.1–80.1), p = 0.003. All the other 19 red cell antigens we tested were not significantly different when stratified according to HIV status.

We also investigated the possible combined influence of the protective and risk-associated blood group antigens on HIV status. In this model, only those blood groups that were significantly associated with HIV infection (C, E, Jk^a^, Lu^b^ and P_1)_ in bivariate analyses were tested. For this purpose, data was subjected to a stepwise logistic regression model. Carriage of the C antigen reduced the odds of HIV infection by about 85%. Simultaneous carriage of the other blood groups did not have a significant effect on HIV status. The results of this analysis are shown in **Table 2**.

**Table 2:** Results of a logistic regression model showing the effect of red cell blood group antigens on HIV status using all five significant antigens.

Antigen	Odds Ratio (95% Confidence Interval)	P-value
E	0.859 (0.169–4.373)	0.855
P_1_	0.428 (0.108–1.693)	0.226
C	0.146 (0.047–0.455)	0.001
Jk^a^	1.001 (0.156–6.408)	1.0
Lu^b^	1.731 (0.314–9.541	0.529

The analysis was further repeated following the systematic removal of the least significant among the covariates. After this stepwise elimination process, only the C, Lu^b^ and P_1_ antigens maintained statistical significance as risk factors in the model. Carriage of P_1_ antigen increased the odds of HIV infection by 2.2 (95% CI 1.1–4.5), p = 0.026 and similarly, carriage of Lu^b^ increased the odds ratio for HIV infection by 2.9 (95% CI 1.5–5.5), p = 0.001. Conversely, presence of C Rh antigen resulted in 40% decrease in HIV risk of infection. The results are shown in **Table 3**.

**Table 3:** Red cell blood group antigens associated with HIV infection in multivariable logistic regression analysis.

Antigen	Odds Ratio (95% Confidence Interval))	p-value
C	0.6 (0.3–1.0)	0.047
P_1_	2.2 (1.1–4.5)	0.026
Lu^b^	2.9 (1.5–5.5)	0.001

We further sought to determine if the frequencies of protective and risk-associated antigens were any different in other populations of African, Caucasian, or Asian ethnicity. The frequency of blood group C and E in this study, though not significantly different from that published for other Blacks (p = 0.3185), were significantly higher in Caucasians or Indians (p<0.05, and p<0.0001, respectively). Northern India had a significantly higher C frequency (p<0.05) than the Botswana population but E was significantly lower in the Northern Indian population (p<0.001). Both Jk^a^ and Lu^b^ were significantly lower in the Botswana population than the other ethnic groups [31–33]. The frequencies for the risk-associated P_1_ was only significantly higher for other Blacks but insignificant for Northern India or Caucasians. A comparison of frequencies of the various antigens according to ethnicity is presented in Table 4.

**Table 4:** Comparative prevalence of risk-associated and risk-lowering blood group phenotypes among Botswana, Northern India and Caucasian ethnicities.

Antigen	Prevalence of antigen in			
	Botswana (This study)	Other Blacks	Northern India	Caucasians
C	29.7%	27%	85%*	68% ***
E	21.2%	22%	17.9%***	29% **
Jk^a^	51.4%	92%***	82.65%***	76% ***
Lu^b^	67.8%	92%***	96.8***	99.8%***
P1	74.6%	94% ***	67.2%	79%

Significance levels:

* P<0.05

** P<0.01

***P<0.001

We did not perform any determination of antigen expression levels for the antigens under investigation. However, assuming that the strength of the antigen-antibody reaction during blood grouping was dependent on the amount of antigen present, we compared weakly reacting individuals (1+) to strongly reacting ones (4+) according to HIV status. Individuals with 4+ reactions for Lu^b^ were indeed more common in the HIV-infected group compared to controls (p<0.0001). Likewise the strongly positive individuals for C occurred with higher frequency among the uninfected controls than the HIV-infected group, suggesting that the observed risk or protection may be related to the density of these antigens on the red cell surface. The frequency of strongly reactive P_1_ in this sub-analysis, though higher among the infected group, was statistically insignificant (p = 0.199).

## Discussion

Blood groups have been implicated as factors in the epidemiology of HIV[1, 17, 22–24, 34]. We studied the distribution of blood groups in Botswana and identified those associated with HIV infection. We therefore determined the frequencies for ABO, Rh, Kidd, Kell, Duffy, Lewis, Lutheran, MNSs, and P_1_ blood group systems in both HIV-infected patients and non-HIV-infected individuals.

The C and E antigens in the Rhesus blood group system were found to be rarer in HIV-infected subjects while that of Jk^a^, Lu^b^ and P_1_ were more prevalent in this group. In comparison to individuals of African ethnicity, the Botswana population does not appear to have any higher frequency of risk-associated blood groups (Jk^a^, Lu^b^ or P_1_) nor does it have any lower frequency of those associated with risk mitigation (C and E). The higher rate of HIV in the Botswana population compared to other Africans is therefore more likely a consequence of other factors than the blood groups herein studied. However, the prevalence of the risk-mitigating antigen C was lower in Blacks (and in Botswana) than in Caucasians or Asians. Given the strength of the influence of the C antigen in the model used, and given the relative rarity of its frequency in the Botswana population, we suspect that the C antigen may possibly play a role in mitigating against HIV-1 infection and may have played a role in the epidemiology of HIV in Botswana.

Our comparison of the effect of these antigens on the HIV-1 subtypes by ethnicity was inconclusive. The frequency of C was lower in Botswana compared to Northern India, which, like Botswana, is experiencing an HIV-1C epidemic. The higher frequency of the protective C and the lower frequency of the risk-associated P_1_ antigens in Northern India may in part explain the lower prevalence of HIV in the Indian population compared to Botswana. However, it was noted that Northern India also had a significantly higher frequency of the risk-associated Lu^b^. It is important, however, to note the multifactorial nature of risk factors for HIV infection, and the fact that the epidemic may not be fully explained on the basis of this limited investigation. This includes the fact that some infections may be compartmentalized within a population due to restricted inter-ethnic interaction. To further appreciate the role of blood groups in the epidemiology of HIV, future studies need to look into the prevalence of these antigens in special groups such as hard-hit families and uninfected high-risk individuals such as female sex workers and discordant couples.

We hypothesized that a higher prevalence of an antigen in the HIV-positive group than the control group possibly indicates that its presence promotes HIV infection, and vice versa. We therefore investigated the potential mitigation of the “protective” phenotypes against the “risky” ones. The odds ratios were therefore computed for risk-associated blood groups in the presence or absence of Rh C or E expression. The presence of C or E did not consistently result in obliteration of the risk-significance in the risk-associated phenotypes. This analysis was also hampered by the simultaneous carriage or alternate carriage of suspect antigens in different individuals, making it difficult to isolate the effect of each antigen.

To further elucidate how the odds ratio is impacted by interaction of all significant blood groups, the data was subjected to a binary logistic regression analysis. The Jk^a^ and E antigens, although highly significant in their association to HIV infection in the isolated Chi-square analysis, failed to maintain significance in the binary logistic regression model. On the other hand, the C, P_1_ and Lu^b^ remained significant with C significantly reducing the odds of HIV infection while P_1_ and Lu^b^ significantly increased the odds of infection.

Our results for blood group O are not in concordance with the reports by other investigators [4, 35] who documented an increased HIV susceptibility for this group. Neither did we find any increased risk in group B individuals as reported in Brazil [34]. There was also no difference in HIV prevalence between D-positive and D-negative subjects as reported in Nigeria [3]. However, we take cognizance of the possible population differences and the effect of other factors such as viral strains in those populations that may account for the disparities observed. But for the Botswana population, the ABO groups do not appear to have any role in enhancing or diminishing HIV risk of infection.

We did not find any risks associated with the Duffy blood group system or its Duffy-null phenotype alluded to elsewhere [17, 21, 22, 36]. Our results in this respect appear to support an insignificant role of Duffy blood group expression consistent with other investigators[20, 37]. However, we did confirm that the Duffy-null phenotype was associated with leukopenia[38], though not neutropenia, in Botswana.

Our findings indicate that P_1_ antigen, a glycosphingolipid, is associated with a higher risk of HIV infection and thus corroborates the role of glycosphingolipids as facilitators of viral fusion to CD4^+^ cells [25, 26]. In their experiments, Puri and colleagues demonstrated that HIV infection of CD4^+^ cells could be averted by addition of a glycosphingolipid inhibitor. Conversely, the presence of the glycosphingolipid increased susceptibility of CD4^+^ cells to HIV infection. The P_1_ antigen is also known to bind several microorganisms including enteropathogenic *Escherichia coli* and parvovirus [14] and has a wide tissue distribution [32], providing multiple opportunities for HIV viral binding, if any. We conclude that our findings corroborate the role of P_1_ as a potential aggravator of HIV infection.

The Lutheran blood group is a member of the immunoglobulin superfamily and has been shown to function as a receptor for laminin [39, 40], a basement membrane protein. The Lutheran blood group also has potential role for signal transduction and is widely distributed in various tissues [39, 41]. Its presence on red blood cells has been implicated in the pathophysiology of sickle cell anemia, where it contributes to vascular occlusion by binding the sickled cells to the vascular endothelium. Dhawan and colleagues reported that HIV-infected monocytes were three-fold more adherent to extracellular matrix than uninfected cells[42], an activity that is facilitated by this blood group[39–41]. This is a crucial step in the extravasation of circulating leukocytes and could shed light on the kinetics of infected macrophages and potential introduction of the virus in remote anatomic sites such as brain and lymph nodes.

The C antigen is an erythrocyte-specific protein with twelve-membrane-spanning domains which is highly hydrophobic and thought to have a role in ammonia transport pathways [43]. An extensive literature search was undertaken to elucidate the mechanism of interaction with HIV. To date no studies have been reported on the association of the molecular structure of Rh C antigen with HIV or other functions that could be linked to HIV infection. We can only speculate that the hydrophobic nature of this antigen may enable it to interact with the viral lipid bilayer by a yet-to-be-explained mechanism. It is hoped that this report will stimulate scientific enquiry in this area to shed light on possible mechanisms.

## Conclusion

We have studied the role of 23 blood group antigens in relation to susceptibility to HIV infection, this being hitherto the most comprehensive study on the role of blood groups in HIV infection. The strongest association in this study was with the Rh antigen C, which also appears to be protective, and is significantly lower in Africans than Caucasians or Indians. On the other hand, there are multiple antigens associated with increased risk that may override the protective role of C.

The protective effect of C or the risk associated with Lu^b^ appears to be related to the strength of the antigen-antibody reactions of these blood group antigens and is likely a function of the density of the antigens on the red cells. A study of the distribution of these antigens in other populations with high rates of HIV infection may shed light on their roles in the HIV pandemic.

## Supporting Information

S1 Table Blood groups raw dataset (0 = Negative, 1 = Positive). (PDF)
