# Supplementary material for: Blood Group Antigens C, Lub and P1 May Have a Role in HIV Infection in Africans
Source: PLoS One. 2016 Feb 22;11(2):e0149883. doi: 10.1371/journal.pone.0149883 (PMC4764295; doi:10.1371/journal.pone.0149883)
Supplement: S1 Table — (PDF) [file pone.0149883.s001.pdf]

P1	M	N	C	c	E	e	Jka	Jkb	A	B	AB	O	D	Lua	Lub	Kpa	Kpb	Fya	Fyb	S	s	Lea	Leb	HIV Status	Duffynull
0	0	1	0	1	0	1			0	1	1	0	1	0	1	0	1	0	0	0	1	0	0	0	1
1	1	0	0	1	1	0			1	1	1	0	1	0	1	0	1	0	1	1	0	0	1	0	
1	0	1	0	1	0	1			0	1	1	0	1	0	1	0	1	1	0	0	1	0	1	0	0
0	1	1	0	1	0	1			1	0	1	0	1	0	1	0	1	0	0	0	1	0	0	0	1
1	1	1	0	1	0	1			1	0	1	0	1	0	1	0	1	0	0	0	1	0	0	0	1
1	1	1	0	1	0	1			1	0	1	0	1	0	1	0	1	0	0	0	1	0	0	0	1
1	1	1	0	1	0	1			0	0	0	1	1	0	1	0	0	0	0	0	1	0	1	0	1
1	1	0	0	1	0	1			0	1	1	0	1	0	1	0	0	0	0	0	1	1	0	0	1
1	1	1	1	1	0	1			0	0	0	1	1	0	1	0	1	0	0	1	1	0	1	0	1
1	1	1	0	1	0	1			0	0	0	1	0	0	1	0	1	1	1	1	1	1	0	0	0
1	1	0	0	1	0	1			0	0	0	1	1	1	1	0	1	0	0	0	1	0	0	0	1
1	1	1	0	1	0	1			0	0	0	1	1	0	1	0	1	0	0	0	1	1	0	0	1
1	1	0	0	1	1	1			1	0	1	0	1	0	1	0	1	0	0	0	1	0	1	0	1
1	1	1	0	1	0	1			1	1	1	0	1	0	1	0	0	0	0	0	1	0	1	0	1
1	0	1	1	1	0	1			0	0	0	1	1	0	1	0	1	0	0	0	1	0	1	0	1
1	1	1	1	1	0	1			0	1	1	0	1	0	0	0	1	0	0	0	1	0	0	0	1
0	1	1	0	1	1	1			0	1	1	0	1	0	1	0	1	0	0	0	1	0	0	0	1
1	1	0	0	1	0	1			0	0	0	1	1	0	1	0	0	0	0	0	1	0	1	0	1
1	1	0	0	1	1	1			0	1	1	0	1	0	0	0	1	0	0	0	1	1	0	0	1
0	0	1	0	1	1	1			0	1	1	0	1	0	0	0	1	0	0	0	1	0	1	0	1
1	1	0	0	1	0	1			1	0	1	0	1	0	1	0	1	0	0	1	1	1	1	0	1
1	1	0	0	1	0	1			0	0	0	1	1	0	1	0	1	0	1		1	1	1	0	0
1	0	1	0	1	0	1			1	1	1	0	1	0	1	0	1	0	0	0	1	1	0	0	1
1	0	1	0	1	0	1			1	0	1	0	1	0	1	0	1	0	0	0	1	0	1	0	1
0	1	0	0	1	0	1			0	0	0	1	1	0	1	0	0	0	0	1	1	0	0	0	1
0	1	1	0	1	0	1			0	0	0	1	1	0	1	0	0	1	0	1	1	0	1	0	0
1	1	0	0	1	0	1			1	0	1	0	1	0	1	0	0	0	0	1	1	0	1	0	1
1	1	1	0	1	1	1			0	0	0	1	1	1	1	0	0	0	0	1	1	0	1	0	1
0	1	1	0	1	0	1			0	0	0	1	1	1	1	0	0	0	0	1	1	0	1	0	1
1	1	1	0	1	0	1			1	0	1	0	1	0	1	0	0	0	0	1	1	1	1	0	1
0	1	1	1	1	1	1			0	1	1	0	1	0	1	0	0	1	1	1	1	1	0	0	0
0	1	1	0	1	1	1			0	0	0	1	1	0	1	0	0	1	0	0	1	1	0	0	0

1	1	1	0	1	0	1			1	0	1	0	1	0	1	0	0	1	1	1	1	0	0	0	0
1	1	0	0	1	0	1			0	1	1	0	1	0	1	0	0	1	1	0	1	0	0	0	0
1	0	1	0	1	1	1			1	1	1	0	1	0	0	0	0	0	1	0	1	1	0	0	0
1	0	1	0	1	0	1			1	0	1	0	1	0	1	0	0	0	0	1	1	0	1	0	1
0	1	1	1	1	0	1			1	0	1	0	1	0	1	0	0	1	0	1	1			0	0
1	0	1	1	1	0	1			1	0	1	0	1		1	1	1		0	0	1			0	
1	1	1	1	0	0	1			0	0	0	1	1	0	1	0	1		1	1	1			0	
1	1	1	1	1	0	1			1	0	1	0	1	1	1	0			1	1	1			0	
1	1	0	1	1	0	1			0	0	0	1	0	0	1	0	1	1	1	1	1			0	0
0	1	0	1	1	0	1			1	0	1	0	1	0	1	0	1	0		1	1			0	
1	1	1	0	1	0	1			0	0	0	1	1		0	0	1			0	1			0	
1	0	1	0	1	1	1			0	0	0	1	1	0	1	0	1			0	1			0	
0	1	0	1	1	0	1			0	0	0	1	1	0	1	0	1		0	0	1			0	
1	1	0	0	1	0	1			1	0	1	0	1	0	0	0	0	0	0	0	1			0	1
1	1	1	1	0	0	1			0	0	0	1	1	0	1	0	1	1	1	1	1			0	0
1	1	1	0	1	1	1			0	0	0	1	1	0	1			0	1	0	1			0	0
1	1	1	0	1	0	1			0	1	1	0	1	0	0	0	1	0	0	0	0	0	0	0	1
1	1	0	0	1	1	1			1	0	1	0	1	0	0	0	1	0	0	0	1	0	0	0	1
1	1	1	0	1	0	1			0	0	0	1	1	0	0	0	1	0	0	0	1	0	0	0	1
1	0	1	1	1	0	1			1	0	1	0	1	0	1	0	1	0	0	0	1	0	1	0	1
0	1	0	0	1	0	1			1	1	1	0	1	0	0	0	1	0	0	1	1	0	1	0	1
1	0	1	0	1	1	1			0	1	1	0	1	0	0	0	0	0	0	0	1	0	0	0	1
1	0	1	0	1	0	1			0	0	0	1	1	0	0	0	0	0	1	1	1	0	1	0	0
1	1	0	0	1	0	1			0	0	0	1	1	0	0	0	0	0	0	1	1	0	0	0	1
1	0	1	1	1	0	1			1	0	1	0	1	0	1	0	1	0	0	0	1	0	0	0	1
1	1	1	1	1	0	1			0	1	1	0	1	0	0	0	1	0	0	0	1	0	0	0	1
1	0	1	0	1	0	1			0	0	0	1	1	0	0	0	1	0	0	1	1	0	1	0	1
1	1	0	0	1	0	1			1	0	1	0	1	0	0	0	1	0	0	0	1	0	1	0	1
1	0	0	0	1	0	1			1	0	1	0	1	0	1	0	0	0	0	1	1	0	0	0	1
1	1	0	0	1	1	0			1	1	1	0	1	0	1	0	0	0	0	0	1	1	0	0	1
1	1	0	0	1	0	1			0	0	0	1	1	0	1	0	0	0	0	0	1	0	0	0	1
1	1	0	0	1	0	1			0	0	0	1	1	0	0	0	1	0	0	1	1	0	0	0	1
1	0	1	1	1	0	1			0	1	1	0	1	0	1	0	1	0	0	0	1	0	0	0	1

1	1	1	0	1	0	1			1	0	1	0	1	1	1	0	0	0	0	1	1	0	0	0	1
1	1	1	1	1	0	1			0	1	1	0	1	0	1	0	1	0	0	0	0	0	0	1	1
0	1	1	1	1	0	1			1	0	1	0	1	0	1	0	1	1	0	1	1	0	0	0	0
1	0	1	0	1	0	1			0	0	0	1	1	0	1	0	1	0	0	1	1	0	0	0	1
1	0	1	1	1	0	1			0	0	0	1	1	0	0	0	0	0	0	0	1	1	1	0	1
1	0	1	1	1	0	1			0	0	0	1	1	0	0	0	1	0	0	0	1	0	0	0	1
1	1	1	1	1	0	1			1	0	1	0	1	0	1	0	0	0	0	0	1	0	1	0	1
0	0	1	0	1	0	1			1	0	1	0	1	0	1	0	0	0	0	0	1	1	0	0	1
1	1	1	0	1	1	1			0	0	0	1	1	0	0	0	0	0	0	0	1	0	0	0	1
0	1	1	1	1	0	1			0	0	0	1	1	1	0	0	0	0	0	0	1	0	1	0	1
1	1	1	1	1	0	1			0	0	0	1	1	0	1	0	0	0	0	0	1	0	1	0	1
1	1	1	0	1	0	1			1	0	1	0	1	0	0	0	0	0	0	1	1	0	0	0	1
1	1	0	0	1	0	1			0	0	0	1	1	0	0	0	0	0	0	0	1	0	1	0	1
1	1	1	1	1	0	1			0	0	0	1	1	0	0	0	0	0	0	0	1	0	0	0	1
1	1	1	1	0	0	1			1	0	1	0	1	0	1	0	0	0	0	0	1	1	0	0	1
1	1	1	0	1	0	1			0	0	0	1	1	0	1	0	0	0	0	1	1	0	1	0	1
1	1	1	0	1	0	1			1	0	1	0	1	0	0	0	0	0	0	1	1	0	0	0	1
0	1	1	0	1	1	1			0	0	0	1	1	0	1	0	0	0	0	0	1	0	1	0	1
1	1	1	0	1	0	1			0	1	1	0	1	0	0	0	0	0	0	0	0	0	0	0	1
1	1	1	0	1	0	1			0	0	0	1	1	0	1	0	0	0	0	0	0	0	1	0	1
1	1	1	1	1	0	1			0	1	1	0	1	0	1	0	0	0	0	0	0	0	0	0	1
0	1	1	0	1	1	1			0	1	1	0	1	0	0	0	0	0	0	0	0	0	0	0	1
1	0	1	0	1	1	1			0	0	0	1	1	0	0	0	0	0	0	0	0	0	0	0	1
1	1	1	0	1	0	1			0	1	1	0	1	0	0	0	0	0	0	0	1	0	0	0	1
0	1	1	0	1	0	1			0	0	0	1	1	0	1	0	0	1	0	0	1	0	1	0	0
1	1	1	1	1	0	1			1	0	1	0	1	0	1	0	0	0	0	1	1	1	0	0	1
1	1	0	0	1	0	1			0	0	0	1	1	0	1	1	0	0	0	0	1	0	0	0	1
1	1	1	0	1	0	1			1	1	1	0	1	0	1	0	0	0	0	0	1	0	0	0	1
1	1	1	1	1	0	1			0	0	0	1	1	0	1	0	1	0	0	1	1	1	0	0	1
1	1	1	0	1	0	1			0	0	0	1	1	0	1	0	1	0	0	1	1	0	1	0	1
1	1	1	0	1	0	1			0	1	1	0	1	0	1	0	0	0	0	0	1	0	0	0	1
0	1	1	0	1	0	1			0	0	0	1	1	0	1	0	0	0	1	0	1	0	1	0	0
1	1	1	0	1	0	1			1	0	1	0	1	0	1	0	0	0	0	1	1	0	1	0	1

0	1	0	0	1	0	1			1	0	1	0	1	0	1	0	0	1	0	1	1	0	0	0	0
0	1	0	0	1	0	1			0	0	0	1	1	0	0	0	1	0	0	0	1	1	0	0	1
1	1	0	0	1	0	1			1	0	1	0	1	1	0	0	0	0	0	1	1	0	0	0	1
0	0	1	0	1	0	1			1	0	1	0	1	0	1	0	0	0	0	0	1	0	0	0	1
0	1	0	0	1	1	1			1	1	1	0	1	0	0	0	0	0	0	1		0	0	0	1
0	1	1	0	1	0	1			0	0	0	1	1	0	1	0	0	0	0	0	1	0	0	0	1
0	1	1	0	1	1	1			0	0	0	1	1	0	0	0	0	0	1	0	1	0	1	0	0
0	1	0	1	1	1	1			1	0	1	0	1	0	1	0	0	0	0	0	1	1	0	0	1
0	0	1	1	0	0	1			1	0	1	0	1	0	1	0	1	1	0	0	1	0	0	0	0
0	1	1	0	1	0	1			1	0	1	0	1	0	1	0	0	0	0	0	1	1	1	0	1
0	1	1	0	1	0	0			0	1	1	0	1	1	0	0	0	0	0	0	1	0	0	0	1
0	1	1	0	1	0	1			1	0	1	0	1	1	0	0	0	0	0	0	1	1	1	0	1
0	1	0	0	1	0	1			0	0	0	1	1	0	1	0	0	0	0	1	1	0	1	0	1
1	1	1	0	1	0	1			0	1	1	0	1									0	0	0	
1	1	0	1	1	1	1			1	0	1	0	1	0	1	0	0	0	1	0	1	0	0	0	0
1	0	1	1	1	0	1			1	0	1	0	1	0	1	0	0	0	0	1	0	0	0	0	1
1	1	1	0	1	0	1			0	1	1	0	1	0	1	0	0	0	1	1	1	0	0	0	0
1	1	0	0	1	0	1			0	0	0	1	1	0	1	0	0	0	0	0	1	1	0	0	1
1	0	1	0	1	1	1			0	1	1	0	1	0	1	0	0	0	0	0	1	1	0	0	1
1	1	1	0	1	0	1			1	0	1	0	1	0	1	0	1	0	0	0	1	1	0	0	1
1	0	1	0	1	0	1			1	1	1	0	1	0	1	0	0	0	0	1	1	0	0	0	1
1	1	1	0	1	0	1			0	1	1	0	1	0	1	0	0	0	0	1	1	0	0	0	1
0	1	0	0	1	0	1			1	1	1	0	1	0	1	0	0	0	0	1	0	0	0	0	1
1	1	0	0	1	0	1			1	0	1	0	1	0	1	0	0	0	0	0	1	0	0	0	1
1	1	0	1	1	0	1			0	0	0	1	1	0		0	0	0	0	0	0	0	1	0	1
0	1	0	0	1	1	1			1	0	1	0	1	0	1	0	0	0	0	0	1	0	0	0	1
1	1	1	0	1	0	1			0	0	0	1	0	1	0	0	0	0	0		1	0	1	0	1
1	1	0	0	1	1	1			0	0	0	1	1	0	1	0	0	0	0	0	1	0	0	0	1
0	1	1	0	1	1	1			0	1	1	0	1	0	0		0	0	0	0	1	0	0	0	1
0	0	1	0	1	1	1			1	0	1	0	1	0	0	0	0	0	0	0	1	0	0	0	1
1	1	1	0	1	1	1			0	0	0	1	1	0	0	0	0	0	0	0	1	0	0	0	1
1	1	1	0	1	0	1			0	0	0	1	1	0	0		0	0	0	0	1	0	0	0	1
1	1	1	0	1	1	1			0	0	0	1	1	0	0	0	0	0	0	1	1	1	0	0	1

1	1	1	0	1	0	1			0	0	0	1	1	0	0	0	0	0	0	1	1	1	0	0	1
1	1	0	0	1	1	1			1	1	1	0	1	0	0	1	0	0	0	1	1	0	0	0	1
1	1	1	1	1	0	1			0	0	0	1	1	0	1	0	0	0	0	1	1	1	1	0	1
1	1	1	1	1	0	1			0	0	0	1	1	0	0	0	0	0	0	1	1	0	1	0	1
1	1	0	0	1	0	1			1	0	1	0	1	0	1	0	0	0	0	0	1	0	1	0	1
1	1	0	0	1	0	1			0	0	0	1	1	0	1	0	0	0	0	1	1	0	1	0	1
1	1	1	1	1	0	1			0	0	0	1	1	0	1	0	0	0	0	0	1	1	0	0	1
1	1	1	0	1	0	1			1	0	1	0	1	0	0	0	1	0	0	1	1	1	0	0	1
1	1	0	1	1	0	1			0	0	0	1	1	0	0	0	0	0	0	1	1	0	0	0	1
1	1	0	1	1	0	1			0	0	0	1	1	0	1	0	0	0	0	1	1	0	0	0	1
0	1	0	0	1	0	1			0	0	0	1	1	0	1	0	0	0	0	1	1	0	1	0	1
1	0	1	0	1	0	1			1	0	1	0	1	0	0	0	1	0	0	0	1	0	1	0	1
0	1	0	0	1	1	1			1	0	1	0	1	0	0	0	0	0	0	0	1	0	1	0	1
1	1	1	0	1	0	1			0	0	0	1	1	0	1	0	0	0	0	1	1	0	1	0	1
1	1	1	1	0	0	1			1	1	1	0	1	0	1	0	0	1	0	0	1	0	0	0	0
1	1	1	0	1	0	1			0	1	1	0	1	0	1	0	0	0	0	0	1	0	0	0	1
1	1	1	1	1	0	1			1	0	1	0	1	0	1	0	0	0	0	0	1	0	0	0	1
0	1	0	0	1	0	1			1	0	1	0	1	0	1	0	0	0	0	0	1	0	1	0	1
0	1	0	1	1	0	1			0	1	1	0	1	0	1	0	0	0	0	0	1	0	0	0	1
1	0	1	0	1	1	0			1	0	1	0	1	0	1	0	0	0	0	0	1	0	1	0	1
1	1	0	1	0	0	1			1	0	1	0	1	0	1	0	0	0	0	0	1	0	0	0	1
1	1	1	0	1	0	1			0	1	1	0	1	0	0	0	0	0	0	0	1	0	0	0	1
0	1	0	0	0	0	1			0	0	0	1	1	0	0	0	0	0	0	1	1	0	0	0	1
1	1	1	0	1	0	1			0	0	0	1	1	0	0	0	1	0	0	0	1	0	0	0	1
1		1	0	1	0	1			0	0	0	1	1	0	0		1	0	0	0	1	0	0	0	1
1	0	1	0	1	1	1			0	0	0	1	1	0	0	0	0	0	0	0	1	0	0	0	1
0	1	0	0	1	1	1			1	0	1	0	1	0	0	0	0	0	0	0	1	0	0	0	1
0	1	1	0	1	0	1			0	1	1	0	1	0	0	0	0	0	0	1	0	0	0	0	1
1		1	0	1	0	1			0	0	0	1	1	0	0	0	1	0	0	0	1	0	0	0	1
0	1	1	0	1	0	1			1	0	1	0	1	0	0	0	1	0	0	1	1	0	0	0	1
1	1	1	0	1	0	1			0	0	0	1	1	0	0	0	1	0	0	0	1	0	1	0	1
0	1	0	0	1	0	1			0	0	0	1	1	0	0	0	0	0	0	1	0	1	0	0	1
0	1	0	1	1	0	1			0	0	0	1	1	0	0	0	1	0	0	0	1	0	0	0	1

0	1	0	1	1	0	1			1	0	1	0	1	0	0	0	1	0	0	0	1	0	0	0	1
0	1	0	0	1	0	1			1	1	1	0	1	0	0	0	1	0	0	0	1	0	0	0	1
1	1	0	0	1	0	1			0	0	0	1	1	0	1	0	1	0	0	0	1	0	1	0	1
1	1	1	1	1	0	1			1	0	1	0	1	0	0	0	1	0	0	0	1	0	0	0	1
1	1	0	0	1	0	1			0	0	0	1	1	0	0	0	1	0	0	0	0	0	0	0	1
1	1	1	0	1	1	1			0	1	1	0	1	0	0	0	1	1	0	0	0	0	0	0	0
1	1	1	0	1	0	1			1	0	1	0	1	0	0	0	1	0	0	0	0	0	1	0	1
0	1	1	1	1	0	1			0	1	1	0	1	0	0	0	1	0	0	0	1	0	0	0	1
0	1	1	0	1	1	1			1	0	1	0	1	0	0	0	0	0	0	0	1	0	0	0	1
0	1	1	0	1	0	1			0	0	0	1	1	0	1	0	0	1	0	0	1	0	0	0	0
1	1	0	0	1	0	1			1	0	1	0	1	0	0	0	1	0	0	0	1	0	0	0	1
1		1	1	1	0	1			0	0	0	1	1	0	0	0	1	0	0	0	1	0	1	0	1
1	1	1	0	1	0	1			0	1	1	0	0	0	0	0	1	0	0	0	1	0	0	1	1
1	1	0	0	1	1	1			0	1	1	0	1	0	1	0	1	0	0	0	1	0	0	0	1
0	0	1	0	1	0	1			0	0	0	1	1	0	1	0	0	0	0	0	1	1	0	0	1
0	0	1	0	1	0	1			1	0	1	0	1	0	0	0	0	0	0	0	0	0	0	0	1
0	1	0	0	1	1	1			0	1	1	0	1	0	0	0	0	0	0	0	1	0	0	0	1
1	0	1	0	1	0	1			1	0	1	0	1	0	1	0	0	0	1	0	1	0	0	0	0
1	1	0	0	1	1	1			1	0	1	0	1	0	0	0	0	0	0	0	1	1	0	0	1
1	0	1	0	1	0	1			0	1	1	0	1	0	1	0	0	0	0	0	1	0	0	1	1
1	1	1	1	1	1	1			0	0	0	1	1	0	0	0	0	0	0	0	1	0	1	0	1
0	1	1	0	1	0	1			0	0	0	1	1	0	1	0	1	0	0	0	1	0	1	0	1
1	1	0	0	1	0	1			0	1	1	0	1	0	0	0	1	0	0	0	1	1	0	0	1
1	0	1	0	1	1	1			1	0	1	0	1	0	0	0	0	0	0	0	1	0	1	0	1
1	1	1	0	1	0	1			0	1	1	0	1	0	0	0	1	0	1	0	1	0	0	0	0
1	1	1	0	1	1	1			0	0	0	1	1	0	0	0	1	0	0	0	1	0	1	0	1
1	0	1	1	1	0	1			1	0	1	0	1	0	1	0	0	0	0	0	1	0	0	0	1
1	0	1	0	1	0	1			0	1	1	0	1	0	1	0	0	0	0	0	1	0	1	0	1
1	1	1	0	1	0	1			1	0	1	0	0	0	1	0	0	0	0	0	1	0	1	0	1
1	1	0	0	1	0	1			1	0	1	0	1	0	1	0	0	0	0	0	1	0	1	0	1
1	1	0	0	1	0	1			0	1	1	0	1	0	1	0	0	0	0	0	1	0	0	0	1
1	1	0	1	1	1	1			1	1	1	0	1	0	1	0	0	0	0	1	1	0	1	0	1
1	1	1	1	1	0	1			1	0	1	0	1	0	1	0	0	0	0	1	1	1	0	0	1

1	1	1	0	1	0	1			0	0	0	1	1	0	0	0	0	0	0	1	1	0	0	0	1
1	1	1	1	1	1	1			1	1	1	0	1	0	1	0	0	1	0	0	1	0	0	0	0
1	1	1	1	1	0	1			1	1	1	0	1	0	1	0	0	0	0	0	1	0	1	0	1
1	0	1	0	1	0	1			0	1	1	0	1	0	1	0	0	0	0	0	1	0	1	0	1
1	1	1	1	1	1	1			1	0	1	0	1	0	1	0	0	0	0	0	1	0	0	0	1
1	1	1	1	1	0	1			1	1	1	0	1	0	1	0	0	0	0	0	1	1	0	0	1
1	1	1	0	1	0	1			1	0	1	0	1	0	1	0	1	0	0	0	1	0	0	0	1
1	1	0	1	1	0	1			1	0	1	0	1	0	1	0	0	0	0	1	1	0	0	0	1
1	1	0	1	1	0	1			0	0	0	1	1	0	1	0	0	0	1	0	1	0	0	0	0
1	1	0	0	1	0	1			1	0	1	0	1	0	1	0	0	0	0	0	1	0	1	0	1
1	1	1	0	1	1	1			0	0	0	1	1	0	1	0	0	0	1	1	1	0	1	0	0
0	1	0	1	1	1	1			0	0	0	1	1	0	1	0	0	0	1	0	1	0	1	0	0
1	0	1	1	1	0	1			0	0	0	1	1	0	1	0	0	1	0	0	1	0	1	0	0
0	1	1	0	1	0	1			1	0	1	0	1	0	0	0	0	0	0	0	1	0	0	0	1
0	1	0	0	1	0	1			1	0	1	0	1	0	1	0	0	0	0	0	1	0	0	0	1
1	1	0	0	1	0	1			0	0	0	1	1	0	1	0	0	0	0	0	1	0	1	0	1
1	1	0	0	1	0	1			0	0	0	1	1	0	1	0	1	0	0	1	1	0	0	0	1
	1	1	0	1	0	1			0	0	0	1	1	0	1	0	1	0	0	0	0	1	0	0	1
	1	1	0	1	0	1			1	1	1	0	0	0	1	0	1	0	0	0	0	0	0	0	1
	1	1	0	1	1	1			0	1	1	0	1	0	1	0	1	0	0	0	0	1	0	0	1
	1	0	0	1	0	1			0	1	1	0	1	0	1	0	0	0	0	0	1	1	0	0	1
	1	1	1	1	1	1			1	1	1	0	1	0	1	0	0	0	0	1	0	0	1	0	1
	0	1	0	1	0	1			0	0	0	1	1	0	1	0	1	0	0	1	0	0	1	0	1
	0	1	0	1	1	1			1	0	1	0	1	0	1	0	0	0	0	1	0	1	1	0	1
	1	1	0	1	0	1			1	0	1	0	1	0	1	0	1	0	0	1	1	0	1	0	1
	1	1	0	1	0	1			1	0	1	0	1	0	1	0	1	0	0	0	0	0	0	0	1
	1	1	0	1	0	1			0	0	0	1	1	0	1	0	0	0	1	1	0	0	1	0	0
	1	1	0	1	0	1			0	0	0	1	0	0	1	0	0					0	0	0	
	1	1	0	1	0	1			0	0	0	1	1	0	1	0	0					0	1	0	
	0	1	0	1	0	1			0	0	0	1	1	0	1	0	0					1	0	0	
	1	0	0	1	0	1			1	0	1	0	1	0	1	0	0					0	0	0	
	0	1	0	1	0	1			1	0	1	0	1	0	1	0	0					0	1	0	
	1	0	1	1	1	1			0	0	0	1	1	0	1	0	0					0	1	0	

	1	1	1	1	0	1			0	0	0	1	1	0	1	0	0					0	1	0	
	0	1	0	1	0	1			1	1	1	0	1	0	1	0	0					0	0	0	
	1	1	0	1	1	1			0	0	0	1	1	0	1	0	0					0	0	0	
	1	1	0	1	0	1			0	0	0	1	1	0	1	0	1					0	0	0	
	1	0	0	1	0	1			0	1	1	0	1	0	1	0	0					0	0	0	
1	1	1	0	1	0	1			1	0	1	0	1	0	1	0	0	0	0	0	1	0	0	0	1
1	1	1	0	1	0	1			0	0	0	1	1	0	1	0	0	0	0	1	1	0	0	0	1
1	0	1	1	1	0	1			0	0	0	1	0	0	1	0	0	1	0	0	1	0	1	0	0
1	1	1	0	1	0	1			1	1	1	0	1	0	0	0	0	0	0	0	1	0	1	0	1
1	1	0	0	1	0	1			1	1	1	0	1	0	0	0	0	0	0	0	1	0	0	0	1
1	1	1	0	1	1	1			1	0	1	0	1	0	0	0	0	0	0	0	1	1	0	0	1
1	1	0	0	1	0	1			1	0	1	0	1	0	1	0	0	0	0	0	1	0	0	0	1
1	0	1	0	1	0	1			1	0	1	0	0	0	1	1	0	0	0	0	1	0	0	1	1
1	1	1	0	1	0	1			0	0	0	1	1	0	1	0	0	0	0	0	1	0	0	1	1
1	1	1	0	1	1	1			0	0	0	1	1	0	1	0	0	0	0	0	1	0	1	1	1
1	0	1	0	1	1	1			0	0	0	1	1	0	1	0	0	0	0	0	1	0	1	1	1
1	1	0	0	1	0	1			0	0	0	1	1	0	1	0	1	0	0	0	1	0	0	1	1
1	1	0	1	1	0	1			1	0	1	0	1	0	1	0	0	0	0	0	1	0	0	1	1
1	1	0	1	1	0	1			0	1	1	0	0	0	1	0	0	0	0	0	1	1	0	1	1
1	1	0	0	1	0	1			0	0	0	1	1	0	1	0	0	0	0	0	1	1	0	1	1
1	1	1	0	1	0	1			1	0	1	0	1	0	0	0	0	0	0	0	1	0	1	1	1
1	1	1	1	1	0	1			0	0	0	1	1	0	1	0	0	0	0	0	1	1	1	1	1
1	1	0	0	1	1	1			1	1	1	0	1	0	1	0	1	0	0	1	1	1	0	1	1
1	1	0	1	1	0	1			1	0	1	0	1	0	1	0	0	1	0	0	1	0	1	1	0
1	1	1	0	1	0	1			0	1	1	0	1	0	0	0	0	0	0	0	1	0	0	1	1
1	1	0	0	1	0	1			0	0	0	1	1	0	0	0	0	0	0	0	1	0	1	1	1
1	1	1	0	1	0	1			1	0	1	0	1	0	0	0	0	0	0	1	1	0	0	1	1
1	1	1	1	1	0	1			1	0	1	0	1	0	0	0	0	0	0	1	1	0	0	1	1
1	1	0	0	1	0	1			0	1	1	0	1	0	0	0	0	0	0	0	1	0	0	1	1
1	1	1	1	1	0	1			0	0	0	1	1	0	0	1	0	0	0	0	1	0	0	1	1
1	1	0	0	1	0	1			0	1	1	0	1	0	0	1	0	0	0	0	1	0	0	1	1
1	1	1	0	1	0	1			1	0	1	0	1	0	0	0	1	0	0	0	1	0	1	1	1
1	1	1	0	1	0	1			1	1		0	1	0	1	0	0	0	1	1	1	0		1	0

1	1	1	1	1	0	1			1	1		0	1	0	1	0	1	0	0	0	1	0		1	1
1	0	1	0	1	0	1			0	1		0	1	0	1	0	0	0	0	0	1	0		1	1
1	1	1	0	1	0	1			0	0		1	1	0	1	0	1	0	0	0	1	0		1	1
1	1	1	0	1	1	1			1	0		0	1	0	1	0	1	0	0	1	1	0		1	1
1	0	1	0	1	0	1			0	0		1	1	0	1	0	1	0	0	0	1	0		1	1
1	1	1	0	1	0	1			0	1		0	1	0	1	0	0	0	0	1	1	0		1	1
1	0	1	0	1	1	1			1	0		0	1	0	1	0	1	0	1	0	1	0		1	0
1	0	1	1	1	0	1			0	0		1	1	0	1	0	1	1	0	1	1	0		1	0
1	1	1	0	1	0	1			0	1		0	1	0	1	0	1	0	0	1	1	0		1	1
1	1	1	1	1	0	1			0	0		1	1	0	1	0	1	1	0	0	1	1		1	0
1	1	0	0	1	0	1			0	0		1	1	0	1	0	1	1	0	1	0	0		1	0
1	1	1	0	1	0	1			0	1		0	1	0	1	0	0	1	0	0	1	0		1	0
1	1	0	0	1	0	1			1	0		0	1	0	1	0	0	0	0	0	1	0		1	1
1	1	0	0	1	0	1			1	1		0	1	0	1	0	1	1	0	0	1	0		1	0
1	0	1	0	1	1	1			0	1		0	1	0	1	0	1	0	0	1	1	0		1	1
1	1	0	0	1	0	1	1	0	1	0		0	1	0	1	0	0	0	0	0	1			1	1
0	0	1	0	1	0	1	1	0	0	0		1	1	0	1	0	0	0	0	0	1			1	1
1	1	0	0	1	0	1	0	0	0	1		0	1	0	1	0	0	0	0	1	1			1	1
1	0	1	0	1	0	1	1	0	0	0		1	1	0	1	0	0	0	0	0	1			1	1
1	1	0	0	1	0	1	0	1	0	0		1	1	0	1	0	0	0	0	0	1			1	1
0	0	1	0	1	0	1	1	0	0	0		1	1	0	1	0	0	1	0	0	1			1	0
0	0	1	1	1	0	1	1	0	0	0		1	1	0	1	0	0	0	0	1	1			1	1
0	1	1	0	1	0	1	1	0	1	0		0	1	0	1	0	0	0	0	0	1			1	1
1	1	0	0	1	0	1	1	0	1	0		0	1	0	1	0	0	0	0	0	1			1	1
0	1	1	0	1	0	1	0	1	0	0		1	1	0	1	0	0	0	0	1	1			1	1
1	1	1	0	1	0	1	0	1	1	0		0	1	0	1	0	0	0	0	1	1			1	1
1	0	1	0	1	0	1	1	0	1	0		0	1	0	1	0	0	0	0	0	1			1	1
0	1	0	0	1	1	1	0	1	1	1		0	1	0	1	0	0	0	0	1	1			1	1
0	1	1	0	1	0	1	1	0	0	0		1	1	0	1	0	0	0	0	1	1			1	1
1	1	0	0	1	0	1	1	1	0	0		1	1	0	1	0	0	0	0	1	1			1	1
1	1	1	0	1	0	1	1	0	0	0		1	1	0	1	0	0	0	1	1	1			1	0
1	1		0	1	0	1	1	0	0	0		1	1				0	0	0	0	1			1	1
0	1		0	1	0	1	1	0	0	0		1	1					0	0	0	1			1	1

1	1		0	1	0	1	1	0	1	0		0	1					0	0	0	1			1	1
1	1		1	1	0	1	1	0	0	1		0	1	0			0	0	1	0	1			1	0
1	1		0	1	0	1	1	0	1	0		0	1	0			0	0	0	1	0			1	1
1	1		0	1	0	1	1	0	0	0		1	1	0			1	0	0	1	1			1	1
1	1		0	1	0	1	1	0	1	0		0	1	0			1	0	1	1	1			1	0
1	1		0	1	0	1	1	1	1	1		0	1	0			1	0	0	0	1			1	1
1	1		0	1	0	1	1	1	1	0		0	1							0	1			1	
1			0	1	0	1	1	0	0	1		0	1							0	1			1	
1			0	1	1	1	1	0	0	1		0	1							0	1			1	
1			0	1	0	1	1	0	0	0		1	1							0	1			1	
1		0	1	1	0	1	1	0	0	1		0	0							0	1			1	
1		1	0	1	0	1	1	1	0	0		1	1							0	1			1	
1		1	0	1	0	1	1	1	0	0		1	1							0	1			1	
1		1	0	1	0	1	1	0	0	1		0	1							0	1			1	
1		0	0	1	0	1	1	1	1	0		0	1							0	1			1	
1		1	0	1	0	1	1	0	1	1		0	0							0	1			1	
1		1	0	1	0	1	1	0	1	0		0	1							0	1			1	
1		1	0	1	0	1	1	0	1	0		0	1							0	1			1	
1		1	0	1	1	1		1	0	0		1	1							0	0			1	
1		0	0	1	0	1	1	0	1	1		0	1							1	1			1	
			1	1	0	1	1	0	0	0		1	1				1			1	0			1	
			0	1	0	1	0	1	1	0		0	1				0			0	1			1	
			0	1	0	1	1	1	1	0		0	1				0			1	1			1	
			0	1	0	1	0	0	0	0		1	1				1			1	1			1	
			0	1	0	1	0	0	0	0		1	1				1			0	1			1	
			0	1	0	1	1	0	0	0		1	1				1			1	1			1	
			0	1	0	1	1	0	0	0		1	1				1			1				1	
			0	1	0	1	1	0	1	0		0	1				1			0				1	
			0	1	0	1	0	1	0	0		1	1							0				1	
			0	1	0	1	0	0	1	0		0	1							0				1	
			0	1	0	1	1	0	0	1		0	1							1				1	
			0	1	0	1	1	1	1	1		0	1							1				1	
			0	1	0	1	1	1	0	0		1	1							0				1	

			0	1	0	1	1	0	0	1		0	0							0				1	
			1	1	0	1	1	0	1	0		0	1							0				1	
			0	1	0	1	1	0	0	0		1	1							1				1	
			0	1	1	1	1	1	0	0		1	1							1				1	
			0	1	1	1	0	1	0	0		1	1							1				1	
			0	1	0	1	0	0	0	0		1	1							1				1	
			0	1	1	1	0	1	1	0		0	1							1				1	
1	1	1	0	1	0	1	1	0	1	0	1	0	1	0	0	0	1	0	1	1	1	0	0	1	0
1	1	1	0	1	0	1	1	0	0	0	0	1	1	0	0	0	1	0	0	0	1	1	0	1	1
1	1	0	1	1	0	1	1	0	1	0	1	0	1	0	1	0	1	0	0	1	1	0	0	1	1
1	0	1	1	1	0	1	1	0	0	1	1	0	1	0	1	0	1	0	0	0	1	0	1	1	1
1	1	1	1	1	1	1	1	1	1	1	1	0	1	0	1	0	1	0	0	0	1	0	0	1	1
1	1	0	0	1	0	1	1	0	0	0	0	1	1	0	1	0	1	0	0	0	1	0	0	1	1
1	1	1	0	1	0	1	1	0	0	0	0	1	1	0	1	0	1	0	0	1	1	0	1	1	1
1	0	1	0	1	1	1	1	0	0	0	0	1	1	0	0	0	1	0	1	0	1	0	1	1	0
1	1	1	0	1	0	1	1	0	0	0	0	1	1	0	1	0	1	0	0	0	1	1	0	1	1
1	1	1	0	1	0	1	1	0	0	0	0	1	1	0	1	0	1	0	0	0	1	0	1	1	1
1	0	1	0	1	0	1	1	0	0	0	0	1	1	0	0	0	1	0	0	0	1	0	0	1	1
1	1	0	1	1	0	1	1	0	0	0	0	1	1	0	1	0	1	0	0	1	1	0	0	1	1
1	1	0	0	1	0	1	1	1	0	0	0	1	0	0	1	0	1	0	0	1	1	0	0	1	1
0	0	1	1	1	1	0	1	0	0	1	1	0	1	0	1	0	1	0	0	0	1	1	1	1	1
1	1	0	0	1	0	1	1	1	1	0	1	0	1	0	1	0	0	0	0	0	1	1	1	1	1
0	1	1	0	1	0	1	0	1	0	0	0	1	1	0	1	0	1	1	0	0	1	1	1	1	0
0	1	0	0	1	0	1	1	0	1	0	1	0	1	0	1	0	0	0	0	1	1	1	1	1	1
1	1	1	0	1	0	1	1	1	0	0	0	1	1	0	1	0	1	0	0	0	1	1	1	1	1
1	1	1	0	1	0	1	1	0	0	0	0	1	1	0	1	0	1	0	0	0	1	1	1	1	1
1	0	1	1	1	0	1	1	0	1	0	1	0	1	0	1	0	1	0	0	0	1	1	1	1	1
1	1	1	0	1	0	1	1	0	0	0	0	1	1	0	1	0	0	1	0	1	1	1	1	1	0
1	1	1	1	1	0	1	1	0	1	0	1	0	1	0	1	0	0	1	1	0	1	1	1	1	0
1	0	1	1	1	0	1	1	1	0	0	0	1	1	1	1	0	0	0	0	0	1	1	1	1	1
1	0	1	0	1	0	1	1	0	1	0	0	0	1	0	1	0	0	0	1	0	1	1	1	1	0
0	1	1	0	1	1	1	1	0	0	0	0	1	1	0	1	0	0	0	0	0	1	1	1	1	1
1	0	1	0	1	0	1	1	1	0	1	1	0	1	0	1	0	0	0	1	0	1	1	1	1	0

1	1	1	0	1	0	1	1	1	0	0	0	1	1	0	1	0	1	0	0	1	1	1	1	1	1
1	1	1	0	1	0	1	1	0	1	0	1	0	1	0	1	0	0	0	0	0	1	0	1	1	1
1		1	0	1	0	1	1	0						0	1	0	0	0	0			0	1	0	1
1		0	1	1	1	1	1	1						0	1	0	1	0	0			1	1	0	1
1		0	0	1	0	1	0	0						0	0	0	0	1	0			0	1	0	0
1		1	0	1	1	1	1	1						0	1	0	1	0	0			0	0	0	1
1		0	0	1	0	1	1	0						0	1	0	0	1	0			1	1	0	0
1		1	1	1	0	1	1	1						0	1	0	1	0	0			1	1	0	1
1		1	0	1	0	1	1	0						0	1	0	1	0	0			0	0	0	1
0		0	1	1	1	1	1	0						0	1	0	1	1	0			1	1	0	0
0		1	1	1	0	1	1	0						0	1	0	1	1	1			1	1	0	0
1		1	1	1	0	1	1	0						0	1	0	1	0	0			0	1	0	1
1		1	0	1	0	1	1	0						0	1	0	1	0	0			0	1	0	1
1		1	1	1	0	1	1	0						0	1	0	1	0	0			1	0	0	1
0		1	1	1	0	1	1	1						0	1	0	1	0	0			1	1	0	1
1		0	1	1	0	1	1	1						0	1	0	0	0	0			1	1	0	1
1		0	0	1	0	1	1	1						0	1	0	0	0	0			1	1	0	1
1		1	1	1	0	1	1	0						0	1	0	0	0	0			1	1	0	1
1		0	0	1	0	1	1	0						0	1	0	0	0	0			1	1	0	1
1		0	0	1	1	1	0	0						0	0	0	0	0	0			1	1	0	1
1		0	1	1	0	1	1	1						0	1	0	0	0	0			1	1	0	1
1		1	1	1	0	1	1	0						0	1	0	0	0	1			1	1	0	0
1		0	0	1	0	1	1	0						0	1	0	1	0	0			1	1	0	1
1		0	0	1	0	1	1	0						0	1	0	1	0	0			1	1	0	1
0		1	1	1	0	1	1	1						0	0	0	1	0	0			1	1	0	1
1		1	1	1	0	1	1	0						0	1	0	1	0	0			1	1	0	1
1		1	1	1	0	1	1	1						0	1	0	1	0	0			1	1	0	1
1		1	0	1	0	1	1	0						0	1	0	1	0	0			1	1	0	1
1		1	1	1	0	1	1	1						0	1	0	0	0	0			1	1	0	1
1		0	1	1	0	1	1	0						0	1	0	0	0	0			1	1	0	1
1		1	1	1	0	1	1	1						0	1	0	1	0	0			1	1	0	1
0		1	1	1	0	1	1	1						0	1	0	1	0	0			1	1	0	1
			0	1	0	1	1	0	0	1	1	0	1	0	1			0	0			0	1	1	1

			0	1	0	1	1	0	0	1	1	0	1	0	1			0	0			0	0	1	1
			0	1	0	1	1	0	0	1	1	0	1	0	1			0	1			0	1	1	0
			0	1	0	1	1	0	0	1	1	0	1	0	1			0	0			1	0	1	1
			0	1	0	1	1	0	0	0	0	1	1	0	1			0	0			1	0	1	1
			0	1	0	1	1	0	0	0	0	1	1	0	1			0	0			1	1	1	1
			0	1	0	1	1	0	0	0	0	1	1	1	1			1	0			0	0	1	0
			0	1	0	1	1	1	0	1	1	0	1	0	1			0	0			0	0	1	1
			1	1	0	1	1	0	1	1	1	0	1	0	1			0	0			0	0	1	1
			0	1	0	1	1	0	0	0	0	1	1	0	1			0	0			0	1	1	1
			0	1	0	1	1	0	0	1	1	0	1	0	1			0	0			1	0	1	1
			0	1	0	1	1	0	1	0	1	0	1	0	1			0	0			0	0	1	1
			0	1	0	1	1	0	1	0	1	0	1	0	1			0	0			0	1	1	1
			0	1	1	1	0	1	1	0	1	0	1	0	1			0	0			0	1	1	1
			0	0	0	1	1	0	0	1	1	0	1	0	1			0	1			0	1	1	0
							1	0	0	1	1	0	1	0	1									1	
							1	0	0	1	1	0	1	0	1									1	
							1	0	0	0	0	1	1	0	0									1	
							1	0	0	0	0	1	1	0	1									1	
							1	0	1	0	1	0	1	0	1									1	
							1	1	0	1	1	0	1	0	1									1	
							1	0	0	1	1	0	1	0	1									1	
							1	0	1	0	1	0	1	0	1									1	
							1	0	0	1	1	0	1	0	1									1	
							1	0	0	1	1	0	1	0	1									1	
							1	0	0	1	1	0	1	0	1									1	
							1	0	0	1	1	0	1	0	1									1	
							0	0	0	0	0	1	1	0	1									1	
							0	0	1	1	1	0	1	0	1									1	
							1	0	0	0	0	1	1	0	1									1	
							0	0																0	
							0	0																0	
							0	0																0	
							0	0																0	

							0	1																0	
							0	0																0	
							0	0																0	
							0	0																0	
							0	0																0	
							0	1																0	
							0	0																0	
							0	0																0	
							0	0																0	
							0	0																0	
							0	0																0	
							0	0																0	
							0	0																0	
							0	1																0	
							0	0																0	
							0	0																0	
							0	0																0	
							0	0																0	
							0	0																0	
							0	0																0	
							0	0																0	
							0	1																0	
							0	0																0	
							0	0																0	
							0	0																0	
							0	0																0	
							0	0																0	
							0	1																0	
							0	1																0	
							0	0																0	
							1	0																0	
							1	0																0	
							0	0																0	

							0	0																0	
							0	1																0	
							0	0																0	
							0	1																0	
							0	1																0	
							1	0																0	
							0	0																0	
							0	0																0	
							0	0																0	
							0	0																0	
							0	0																0	
							0	0																0	
							0	1																0	
															0		0	0					1	1
															0		0	0					1	1
															0		1	0					1	0
															0		0	0					1	1
															0		0	0					1	1
															0		0	0					1	1
															0		0	0					1	1
															0		0	0					1	1
															0		0	0					1	1
															0		0	0					1	1
															0		0	0					1	1
															0		1	0					1	0
															0		0	0					1	1
															0		0	0					1	1
															0		0	0					1	1
															0		0	0					1	1
															0		0	0					1	1
															0		0	0					1	1
															0		0	0					1	1
															0		1	0					1	0

																0		1	0					1	0
																0		0	0					1	1
																0		0	0					1	1
																0		0	0					1	1
																0		0	0					1	1
																0		0	1					1	0
																0		0	0					1	1
																0		0	1					1	0
																0		0	0					1	1
																0		0	0					1	1
																0		0	0					1	1
																0		0	0					1	1
																0		0	0					1	1
																0		0	0					1	1
																0		0	1					1	0
																0		0	0					1	1
																0		0	1					1	0
																0		0	1					1	0
																0		0	1					1	0
																0		0	0					1	1
							1	0																0	
							1	0																0	
							1	1																0	
							1	0																1	
							1	0																0	
							1	1																0	
							1	0																0	
							0	0																0	
							1	1																0	
							1	0																0	
							1	0																0	
							1	1																0	
							0	0																0	

							0	1																0	
							1	0																0	
							0	0																0	
							1	1																0	
							1	1																0	
							1	1																0	
							1	1																0	
							1	0																0	
							1	0																0	
							1	1																0	
							1	0																0	
							1	1																0	
							1	0																0	
							1	0																0	
							1	1																0	
							1	0																0	
							1	0																0	
